# Alexithymia Profile in Relation to Negative Affect in Parents of Autistic and Typically Developing Young Children

**DOI:** 10.3390/brainsci10080496

**Published:** 2020-07-29

**Authors:** Elisa Leonardi, Antonio Cerasa, Francesca Isabella Famà, Cristina Carrozza, Letteria Spadaro, Renato Scifo, Sabrina Baieli, Flavia Marino, Gennaro Tartarisco, David Vagni, Giovanni Pioggia, Liliana Ruta

**Affiliations:** 1 Institute for Biomedical Research and Innovation (IRIB), National Research Council of Italy, 98164 Messina, Italy; elisa.leonardi@istitutomarino.it (E.L.); antonio.cerasa@irib.cnr.it (A.C.); francescaisabella.fama@istitutomarino.it (F.I.F.); cristina.carrozza@istitutomarino.it (C.C.); spadarolia@gmail.com (L.S.); flavia.marino@irib.cnr.it (F.M.); gennaro.tartarisco@irib.cnr.it) (G.T.); david.vagni@irib.cnr.it (D.V.); giovanni.pioggia@irib.cnr.it (G.P.); 2 S. Anna Institute and Research in Advanced Neurorehabilitation (RAN), 88900 Crotone, Italy; 3 Centre for Autism Spectrum Disorders, Child Psychiatry Unit, Provincial Health Service of Catania (ASP CT), 95100 Catania, Italy; renato.scifo@aspct.it (R.S.); sabrina.baieli@aspct.it (S.B.)

**Keywords:** autism, alexithymia, anxiety, depression, TAS-20, TSIA, parents, broader autism phenotype

## Abstract

In our study, we explored the construct of alexithymia in parents of children with and without ASD using a multi-method approach based on self-rated and external rater assessment. We also assessed the level of self-report measures of negative affect states such as trait anxiety and depression, and investigated the correlation between the alexithymia construct, trait anxiety, and depression within the broader autism phenotype (BAP). A total sample of 100 parents (25 mothers and 25 fathers in each group) were administered the TAS-20 and the TSIA to measure self-reported and observer-rated alexithymia traits, as well as self-report measures of anxiety and depression. Study results showed that the TSIA but not the TAS-20 was able to detect significant group differences in alexithymia traits among parents of children with and without ASD, with parents of ASD children displaying significantly higher levels of alexithymia. Furthermore, differently from the TAS-20, no significant correlations between the TSIA and measures of anxiety and depression were detected. Taken together, our results suggest the importance of using multi-method approaches to control for potential measurement bias and to detect psychological constructs such as alexithymia in subclinical samples such as parents of children with ASD.

## 1. Introduction

Alexithymia literally means “absence of words for emotions”. It is a personality construct, normally distributed in the general population [1,2], characterized by deficits identifying and describing one’s own emotions and feelings, problems distinguishing between feelings and bodily sensations of emotional arousal, lack of fantasy, externally oriented cognitive style, and impairment in cognitively mapping their feeling states onto internal bodily responses [3,4,5,6]. Difficulties in emotional awareness may have a negative impact on subjective emotion regulation [7] and, in turn, may compromise the understanding of others’ emotions, giving rise to problems in social interaction. It has been reported, indeed, that individuals with alexithymia show difficulties distinguishing and appreciating emotions expressed by others, and consequently, they are likely to show nonempathic and idiosyncratic socio-emotional responses [8]. Deficits in empathy, specifically in the cognitive component of recognizing and understanding others emotions, have also been reported as one of the distinctive features of autism spectrum disorders (ASD) [9,10,11,12,13], a neurodevelopmental condition characterized by socio-communicative impairments and restricted and repetitive patterns of behaviors and interests [14]. A significant association between ASD and alexithymia has been reported, with at least half of individuals with ASD experiencing co-occurring alexithymia [15,16,17,18,19]. The interrelationship between the two conditions is complex, and it is still debated whether alexithymia and ASD share common etiological roots or if, instead, alexithymia is a symptomatological manifestation within the ASD neuropsychological functioning [20]. Some broad genetic and neurobiological overlapping between the two conditions have been reported, including the involvement of the serotonin and oxytocin systems, and brain areas related to emotion processing, specifically the amygdala, cingulate, and prefrontal cortex [21]. It has been suggested that a common genetic vulnerability between autism and alexithymia could lead to atypical brain networks, associated in turn with different behavioral patterns that manifest themselves mainly with symptoms of alexithymia, autism, or both [22,23]. At the behavioral level, the intersection between alexithymia and ASD is equally controversial and questions the role of alexithymia in relation to emotion recognition and interoception (the ability to interpret the internal state of the body). Indeed, the abilities to perceive one’s own emotions and bodily signals are closely interrelated in social-emotional development [24]. Some studies, comparing individuals with and without ASD on alexithymia, have found that alexithymia, rather than autism per se, predicts difficulties in facial emotion recognition [17], while other evidence in nonclinical samples reported that autism traits more than alexithymia were a stronger predictor of atypical empathy [25]. In relation to interoception, across clinical and nonclinical samples, alexithymia, but not autism, has been associated with impaired interoception [23,24]. Furthermore, alexithymia associated traits have also been reported in relatives of individuals with ASD [26] who show subclinical autism traits, reported as the broader autism phenotype (BAP) [27,28,29,30,31]. The BAP is characterized by below the diagnostic threshold social and cognitive deficits, restricted behavior patterns, ASD-like personality characteristics, and psychiatric difficulties in relatives of autistic individuals [28,29,30,31,32,33,34,35,36,37]. Furthermore, higher levels of alexithymia in fathers of children with ASD were related to higher severity on the repetitive behavior domain in their children [38]. The most widely used questionnaire to evaluate alexithymia is the Toronto Alexithymia Scale (TAS-20) [39]. The TAS-20 is a self-report measure consisting of 20 items rated on a five-point Likert scale. The higher the score on the TAS-20, the greater the alexithymia traits. Although consistent empirical evidence over the past 25 year period has confirmed the reliability and validity of the TAS-20 to tap into the alexithymia construct [40], some concerns about applying a self-report measure to individuals who have difficulties identifying their own emotional states and lack the appropriate insight on this impairment, have been raised. Self-assessment of the interoceptive deficit in alexithymia would, in fact, imply a self-reliance bias being that the main characteristic of alexithymia is exactly the absence of recognition of one’s feelings and bodily sensations [19,41,42,43]. Another shortcoming of the TAS-20 is that the measure does not include the dimension of the imaginative processes, which represents one of the facets of the complex alexithymia construct. Furthermore, scores at the TAS-20 have been correlated with negative affect states, particularly depression and anxiety. This association may be partially explained by a neuropsychological overlap between these dimensions, being that impairment in interoception is frequently associated with negative affect [44], but might also be related to a negativity response bias due to the self- report nature of the TAS-20 [45,46,47]. To overcome this limitation, in 2006, Bagby, Taylor, and Parker developed the Toronto structured interview for Alexithymia (TSIA) [48], a structured interview consisting of 24 questions and carried out by an external examiner who scores the items based on the individual responses across four core domains such as difficulty identifying feelings (DIF), difficulty describing feelings (DDF), externally oriented thinking (EOT), and imaginal processes (IMP). Several studies have confirmed the internal and convergent validity as well as cultural stability of the TAS-20 and the TSIA in nonclinical populations, as well as clinical patients, and have suggested that the TSIA is able to more reliably assess the “fantasy” facet of the alexithymia construct and to better disentangle negative affect states such as depression and anxiety that may partially overlap with the alexithymia construct [48,49,50,51,52,53].

To the best of our knowledge, the only study assessing alexithymia in family members using the TSIA was conducted in parents of anorexic daughters [54], reported significantly higher levels of alexithymia using the observer-rated measure as compared to the self-report measure in parents of the clinical group. Also, as far as we are aware, no studies assessing the alexithymia profile through both self-report and interviewer-administered measures in relatives of ASD individuals have been conducted. Hence, in our study, we used both the TAS-20 and the TSIA to investigate alexithymia in parents of children with and without ASD. Furthermore, since it has been demonstrated an association between alexithymia as measured by the TAS-20 and negative affect states, a secondary goal of the study is to explore the association between both self-report and rater-assessed alexithymia scores and measures of depression and trait anxiety. Previous findings have reported, in fact, significantly higher self-reported levels of trait anxiety and depression symptoms in parents of children with ASD [55,56,57,58,59,60] and it may be relevant to investigate the overlapping alexithymia construct in relation to these dimensions within the BAP trying to disentangle potential biases related to the administration method.

We expect that parents of ASD children, compared to parents of typically developing children, present higher levels of alexithymia at both the TAS-20 and the TSIA, and that the TSIA, in particular, will be able to keep aside co-occurrent traits of depression and anxiety.

## 2. Methods

### 2.1. Participants

Parents were enrolled and tested in the context of an ongoing study on young children with and without ASD, aged between 3 and 6 years of age, both males and females (30% females in the ASD group and 48% females in the TD group). Parents of children with ASD were recruited and tested at the clinical facilities of the Institute for Research and Innovation in Biomedicine of the National Research Council of Italy (IRIB-CNR) in Messina and at the Centre for Autism Spectrum Disorders, Child Psychiatry Unit, Provincial Health Service (ASP-CT) in Catania, Italy. Parents of typically developing children (TD) were recruited and tested in three different mainstream nursery schools and primary schools in Messina. Inclusion criteria in both the ASD and TD parent groups were: (1) biological parents; (2) native Italian speakers; (3) between the age of 25 and 55 years. Specific inclusion criterion in the ASD parent group was having a child with a confirmed clinical diagnosis of ASD according to the Diagnostic and Statistical Manual of Mental Disorders, Fifth Edition [14], while exclusion criteria in the TD parents group were having a child with a clinical diagnosis of neurodevelopmental conditions (such as language and/or motor delay, ADHD, etc.) and a family history of intellectual disabilities, language delay and ASD. A total sample of 112 parents were recruited. According to the inclusion and exclusion criteria, 12 parents (8 parents in the TD group and 5 parents in the ASD group) were excluded for the following reasons: four couples of parents (2 couples in each group) were adoptive parents; two couples of parents of TD children had a first degree relative with ASD; one parent (mother) in the TD group presented a mild intellectual disability; one parent (mother) in the ASD group was a foreigner and had significant difficulties with understanding written and spoken Italian language and finally 4 couples of parents (3 couples in the TD group and 1 couple in the ASD group) who initially agreed to participate, subsequently refused to contribute to the study. A final sample of 50 parents (25 mothers and 25 fathers) of ASD children and 50 parents of TD children (25 mothers and 25 fathers) were fully tested and analyzed in the study.

The study received ethical clearance by the Ethics Committees of CNR (ethical clearance, 01.08.2018) and ASP-CT (Prot. N. 498), respectively, and all the caregivers signed an informed consent to participate in the study.

### 2.2. Measures and Procedures

All enrolled subjects completed the following neuropsychological assessment battery: (1) the State Trait Anxiety Inventory-Form Y (STAI-Y), a self-report questionnaire which consists of 20 questions scored on a 4-point Likert-type scale, ranging from 1 to 4 (from “almost never” to “almost always”). The total score at the STAI-Y ranges from 20 to 80, with scores >40 indicating above-average levels of anxiety. (2) The Beck Depression Inventory (BDI-II) is a self-report questionnaire with 21 items, rated on a 4-point scale from 0 to 3, based on the diagnostic criteria for depressive disorders. Inventory cutoffs are: 0–13: minimal depression, 14–19: mild depression, 20–28: moderate depression, and 29–63: severe depression [61,62]; (3) The Toronto Alexithymia Scale (TAS-20) [39] is a self-report scale comprised of 20 items. Each item is rated on a five-point Likert scale ranging from 1 (“strongly disagree”) to 5 (“strongly agree”). The TAS-20 is a reliable and valid measure of emotion processing in adults and includes a total score and three subscales: Difficulty Identifying Feelings (DIF), Difficulty Describing Feelings (DDF), and Externally-Oriented Thinking (EOT). The empirically derived cutoff score of 61 is used for identifying individuals with ‘‘high’’ versus ‘‘low’’ alexithymia. (4) The Toronto Structured Interview for Alexithymia (TSIA) is a structured interview with prompts and probes which incorporates the same three subscales included in the TAS-20 (DIF, DDF, and EOT, respectively), and an extra subscale investigating Imaginal Processes (IMP). The TSIA consists of 24 items, six items for each of the four salient facets of the alexithymia construct; (5) the “Reading the Mind in the Eyes” Test Revised (Eyes Test) [63]. In the Eyes Test, the participant is presented with a series of 36 black and white photographs of the eye-region of different actors and actresses and is asked to choose which of four words best describes what the person in the photograph is thinking or feeling. We implemented the test on an iPad tablet 9.7 in and collected both accuracy (number of correct answers) and reaction time (RT = length of time taken to answer in seconds); (6) the Wechsler Abbreviated Scale of Intelligence Second Edition (WASI-II) [64] to assess the intelligent quotient (IQ) in all the participants. All questionnaires were administered in a standardized way and in the same sequence to all participants. Assessment administration took about 2 h and was split into two sessions over two consecutive days. In the first session, participants filled in the TAS-20, the STAI-Y and the BDI-II, while during the second visit, the TSIA and the WASI-II were administered by an experienced clinical neuropsychologist (E.L.), specially trained on the administration procedures and scoring of the measures.

### 2.3. Statistical Analysis

Statistical analyses were performed using SPSS Version 23.0. Kolmogorov–Smirnov test was carried out and confirmed the assumptions of normality for all variables. A chi-square test was used for categorical variables, while the Mann–Whitney U-test was run for ordinal variables. An independent two-sample *t*-test was used to analyze between-group differences for continuous, normally distributed variables. The *t*-test’s effect size was calculated using Cohen *d*, where an effect size value of 0.2 is considered a small effect, of 0.5 is considered a medium effect, and values of 0.8 and above are considered large effects. Pearson’s correlations between TAS-20 and TSIA total scores and their dimensions, correlations between TAS-20 and TSIA and correlations of TSIA and TAS-20 with BDI-II and STAI-Y2 respectively, were performed.

All statistical analyses were 2-tailed; α levels were corrected for multiple comparisons using hierarchical Sidak correction. We used two primary measures (TAS-20 and TSIA) for fathers’ and mothers’ questionnaires respectively, therefore, we had 4 primary variables, and we set α_c_ < 0.013. Only if the total score is significant, we analyzed the sub-scales for each measure. TAS-20 has 3 sub-scales and TSIA has 6 sub-scales, therefore α_c_ < 0.017 for the first sub-scales analysis and α_c_ < 0.009 for the second one. The Eyes Test accuracy and reaction Time were added as secondary measures of the BAP and α_c_ < 0.01 was set. In the ancillary correlation analyses, we used a similar procedure setting α_c_ < 0.003 for the correlation among Eyes Test, STAI-Y and BDI-II with TAS-20 and TSIA total scores, α_c_ < 0.009 and α_c_ < 0.004 for the correlation among Eyes Test, STAI-Y and BDI-II with TAS-20 and TSIA sub-scales.

## 3. Results

Table 1 shows the demographic and clinical characteristics of the sample. Parents of children with and without ASD did not differ as regards to descriptive variables and IQ. Parents on the ASD group were characterized by significantly higher levels of trait anxiety (both for mothers and fathers) and depression (more evident in the mothers) with respect to TD parents. Furthermore, mothers of ASD children were significantly slower than mothers of TD children in recognizing emotions from the eyes. Inter-correlations among TAS-20 and TSIA with their respective subscales were all statistically significant (all *p*-values < 0.001) and correlations ranged from moderate to high (from r = 0.59 to r = 0.80 for the TAS-20 and from r = 0.65 to r = 0.82 for the TSIA in the ASD parent group and from r = 0.66 to r = 0.77 for the TAS-20 and from r = 0.61 to r = 0.73 for the TSIA in the TD parent group). The TAS-20 demonstrated also a modest association with the TSIA (r = 0.40; *p* = 0.001 and r = 0.44; *p* = 0.001 in the ASD and TD parent group respectively), confirming convergent validity of the two measures. On the TAS-20 total score and subscales, no significant between-group difference has been detected, neither in the mothers nor in the fathers (Table 2). Conversely, using the TSIA, we found a significant group difference on the total score, with parents in the ASD group scoring significantly higher (i.e., demonstrating more alexithymia traits) than parents in the TD group (*p* = 0.003 and *p* = 0.0001 for mothers and fathers respectively). Furthermore, investigating the TSIA subdomains, mothers on the ASD group scored significantly higher in the “operative thinking (OT)” subscale (*p* = 0.004), while fathers on the ASD group, showed significantly higher scores in all the subscales apart from the “difficulty identifying feelings (DIF)” subscale (all *p*’s < 0.009). When we analyzed the correlations between the TAS-20 and the TSIA (and their subscales) with the negative effect self-report measures (STAI-Y and the BDI-II) separately in the ASD and TD parent samples, additional significant findings were detected. In the ASD parent group, measures of anxiety and depression were significantly associated to the TAS-20 total scores in both mothers (r = 0.58, *p* < 0.0001 and r = 0.61, *p* < 0.0001 respectively) and fathers (r = 0.37, *p* = 0.02 and r = 0.36, *p* = 0.03 respectively). However, in the ASD father group, statistical significance did not survive correction for multiple comparisons. Comparable results were obtained in the TD parent group, with a significant positive association between the TAS-20 total scores and the STAI-Y and BDI-II scores in both mothers (r = 0.45, *p* = 0.002 and r = 0.36, *p* = 0.01 respectively) and fathers (r = 0.51, *p* < 0.0001 and r = 0.31, *p* = 0.03 respectively). However, in both mothers and fathers, correlations between the TAS-20 and the BDI-II did not survive the threshold for multiple comparisons. Conversely, we did not observe any significant relationship between the TSIA total scores and measures of anxiety and depression in neither group. Correlations between the alexithymia scales (and subscales) and the anxiety and depression measures, corrected for multiple comparisons, are reported in Table 3 and Table 4. As an ancillary analysis, to explore if the group difference on the Eyes Test RT in mothers was driving the group differences found on alexithymia, anxiety, and depression, we analyzed the relationship between these measures in the ASD group. We found no significant correlations between the Eyes Test RT and the other measures (Eyes Test RT vs. TAS-20 total score: r = −0.13, *p* = 0.3; vs. TSIA total score: r = −0.12, *p* = 0.4; vs. STAI-Y: r = −0.06, *p* = 0.6; vs. BDI-II: r = −0.16, *p* = 0.2).

## 4. Discussion

In our study, we explored the construct of alexithymia in parents of children with and without ASD using a multi-method approach based on self-rated and external rater assessment. Both the TAS-20 and the TSIA were administered to all the participants showing that the TSIA but not the TAS-20 was able to detect significant group differences in alexithymia traits among parents of children with and without ASD, with parents of ASD children displaying significantly higher levels of alexithymia as reflected by the total score and the subscale scores, especially for fathers. This result suggests that the assessment method has an impact on the capacity to detect alexithymic traits in subclinical populations such as parents of ASD children. The TAS-20, in fact, implies that people are aware and able to identify and describe their own feelings and strongly relies on the self-perception of the subject. Individuals with higher alexithymia traits may lack awareness of their own difficulties because of their meta-emotional impairment [65], and a structured interview such as the TSIA may possibly avoid this bias, as the examiner can use probes and examples to more in-depth assess the presence and degree of alexithymia. This result is in line with previous evidence, in a different clinical sample, reporting that parents of anorexic daughters display significantly higher alexithymia traits at the TSIA as compared to the TAS-20 [54]. Furthermore, we explored the dimensions of negative affect and trait anxiety in our sample of parents, and in agreement with previous studies, we found significantly higher levels of self-reported trait anxiety and depression in parents of children with ASD (both in mothers and fathers), compared with parents of TD children. Last but not least, we analyzed the correlation between measures of alexithymia and self-report measures of depression and trait anxiety to investigate if negative affect may influence rater’s answers to the TAS-20. We found that the TAS-20 total score and the subscales “difficulty identifying feelings (DIF)” and “difficulty describing feelings (DDF)”, but not the TSIA total and subscale scores, were strongly associated with measures of both anxiety and depression in mothers of ASD children. Similar results were observed in the TD parent group, with the TAS-20 being strongly associated with anxiety traits and depression, especially in fathers, and the TSIA showing no significant correlations (apart from the subscale “affect awareness” which was associated with depression in mothers). These findings may suggest that symptoms related to negative emotional states such as reduced cognitive flexibility, emotional awareness, and affective range may influence self-rated responses on the TAS-20. Co-occurring anxiety and depression, in fact, may affect the way one’s emotions are self-judged and instead, having a structured interview such as the TSIA, may ensure a more objective and reliable assessment of the facets related to alexithymia reducing the potential misinterpretation of one’s emotional state due to self-judgment in relation to negative affect. In line with our results, previous evidence reported that negative affectivity influences the rater’s self-report on the TAS-20 items, especially on the DIF and DDF factor subscales, while no association between the TSIA scores and scores at the STAI-Y and the DBI-II was found [46,49,53].

Finally, to explore if aspects of the broader autism phenotype (BAP) in the ASD parent group would account for the results obtained on the alexithymia and negative affect dimensions, we used the Eyes Test to measure emotion recognition and Theory of Mind (ToM) abilities in both groups. Previous evidence, reported impairment in emotion recognition at the Eyes Test in parents of ASD children [66]. We found that mothers of ASD children were significantly slower than mothers of TD children in detecting emotional, mental states from eyes, but no relationship between this measure and measures of alexithymia, anxiety, and depression was found, suggesting that in our ASD parent sample, atypical empathy is not associated to alexithymia traits nor to negative affectivity. Our results, in the light of the mixed findings reported in the literature about the influence of alexithymia on atypical empathy [17,25,67], clearly indicate that measuring these two constructs in relation to autism traits is not simple and generalization from clinical samples to sub-clinical samples such as parents of ASD children to the general population poses complex conceptual and methodological issues that should be taken into account.

Some limitations should be considered with regard to the study and the use of the TSIA. Firstly, the study sample size was relatively small, although rigorously selected according to precise inclusion and exclusion criteria to avoid sampling bias; furthermore, there was no opportunity to conduct a specific psychometric and clinical evaluation on the TD children, to exclude not diagnosed neuropsychiatric conditions, which may have influenced the responses in the TD parent group. Finally, in relation to the use of the TSIA, it should be considered that the time needed to complete the interview, as well as the need for appropriate training in administration procedures and scoring, make the use of the TSIA still limited and more difficult in clinical practice. Further studies are warranted to replicate these results and to generalize these findings from clinical samples to the general population.

## 5. Conclusions

Our study explored the construct of alexithymia in parents of children with and without ASD using a multi-method approach, and also investigated the correlation between the alexithymia construct, trait anxiety, and depression within the broader autism phenotype (BAP). Based on our findings, in subclinical populations such as parents of children with ASD, the use of an external-rater assessment should be considered to allow a more accurate detection of the presence and degree of alexithymia and to disentangle potential influences of negative affect states and trait anxiety on the alexithymia dimension.

## Figures and Tables

**Table 1:** Descriptive and neuropsychological characteristics in parents of ASD and TD children.

	ASD (*n* = 50)	TD (*n* = 50)	*p*-Level	d’s Cohen
Age Mother	38.3 ± 4.8	39.9 ± 5.3	0.31 *	-
Age Father	41.7 ± 8.4	43.2 ± 5.2	0.95 *	-
Education Mother	3 [2–5]	5 [2–6]	0.05 °	-
Education Father	3 [2–6]	3 [2–6]	0.66 °	-
Verbal IQ Mother	97.8 ± 8.9	98.8 ± 9.9	0.67 *	0.11
Performance IQ Mother	102.1 ± 12.5	101.7 ± 13.9	0.91 *	0.02
Total IQ Mother	100.1 ± 10.3	104.7 ± 22.7	0.3 *	0.26
Verbal IQ Father	97.9 ± 8.1	95.6 ± 8.3	0.31 *	0.28
Performance IQ Father	101.7 ± 12.2	105.7 ± 12.2	0.23 *	0.32
Total IQ Father	103.5 ± 23.8	104.4 ± 8.4	0.53 *	0.05
STAI-Y Mother	50.3 ± 10.9	43.9 ± 7.8	0.002 *	0.68
STAI-Y Father	49.5 ± 9.6	44.8 ± 7.9	0.01 *	0.54
BDI-II Mother	11.4 ± 8.8	7.2 ± 5.9	0.01 *	0.56
BDI-II Father	7.6 ± 6.3	4.8 ± 4.6	0.03	0.51
Eyes Test Accuracy Mother	22.6 ± 4.2	22.4 ± 4.4	0.93	0.02
Eyes Test RT Mother	328.3 ± 185.2	230.2 ± 82.7	0.005	0.68
Eyes Test Accuracy Father	20.8 ± 4.1	22.1 ± 3.5	0.23	0.31
Eyes Test RT Father	257.8 ± 88.6	247.3 ± 49.9	0.59	0.14

Data are given as mean values (SD), percentage or median [range] as appropriate. ^§^ chi-square test; ° = Mann–Whitney U-test; * = Two sample *t*-test. ASD, autism spectrum disorders; TD, typical development; education labels, 1 = primary school; 2 = secondary school; 3 = high school; 4 = bachelor degree; 5 = master degree; IQ, intelligent quotient; STAI-Y, State Trait Anxiety Inventory-Form Y; BDI-II, Beck depression inventory; eyes test accuracy, number of correct answers; eyes test RT, length of time taken to answer in seconds.

**Table 2:** Alexithymia profile in parents of ASD and TD children.

	ASD	TD	*p*-Level *	*d*’s Cohen
	(Mean ± SD)	(Mean ± SD)		
**Toronto Alexithymia Scale (TAS-20)**				
**Total score Mother**	39.6 ± 12.3	39.1 ± 9.5	0.79	0.05
DIF Mother	12.1 ± 5.5	11.4 ± 4.8	0.53	0.13
DDF Mother	10.3 ± 4.6	10.3 ± 4.5	0.97	0.01
EOT Mother	17.5 ± 4.2	17.1 ± 4.3	0.65	0.09
Total score Father	42.3 ± 10.8	40.9 ± 9.9	0.53	0.13
DIF Father	11.4 ± 5.2	11.4 ± 4.7	0.99	0.01
DDF Father	11.4 ± 4.4	10.6 ± 9.1	0.38	0.11
EOT Father	19.4 ± 4.7	19.1 ± 3.9	0.68	0.07
**Toronto Structured Interview for Alexithymia (TSIA)**				
DIF Mother	1.6 ± 1.5	0.9 ± 0.7	0.02	0.6
DDF Mother	2.2 ± 2.3	1.6 ± 1.9	0.22	0.29
EOT Mother	2.9 ± 1.9	2.1 ± 1.7	0.04	0.44
IMP Mother	3.8 ± 1.9	2.7 ± 2.0	0.02	0.56
AA Mother	3.8 ± 3.0	2.5 ± 2.4	0.04	0.48
OT Mother	6.7 ± 2.9	4.8 ± 2.5	**0.004**	**0.71**
Total score Mother	10.5 ± 4.6	7.3 ± 4.4	**0.003**	**0.71**
DIF Father	2.3 ± 2.6	1.0 ± 1.6	0.04	0.6
DDF Father	3.8 ± 3.3	1.3 ± 1.5	**0.001**	**0.97**
EOT Father	4.2 ± 2.8	2.3 ± 1.5	**0.006**	**0.85**
IMP Father	4.2 ± 2.3	2.1 ± 1.2	**0.001**	**1.15**
AA Father	5.9 ± 5.3	2.3 ± 2.4	**0.004**	**0.87**
OT Father	8.5 ± 4.5	4.4 ± 2.1	**0.0001**	**1.16**
Total score Father	14.9 ± 9.1	6.7 ± 3.7	**0.0001**	**1.19**

DIF, difficulty identifying feelings; DDF, difficulty describing feelings (DDF); OT, externally-oriented thinking; IMP, imaginal processing; AA, affect awareness; OT, operative thinking. * α levels are corrected for multiple comparisons using hierarchical Sidak correction. An α_c_ < 0.01 was set for the TAS-20 total score and subscales and for the TSIA total score and an α_c_ < 0.009 was set for the TSIA subscales.

**Table 3:** Correlations between alexithymia scales, trait anxiety, and depression in parents of ASD children.

ASD Parent Group	STAI-Y	STAI-Y	BDI-II Mother	BDI-II Father
	Mother	Father		
**Toronto Alexithymia Scale (TAS-20)**				
**Total score Mother**	*r* = 0.58		*r* = 0.61	
	*p* < *0*.0001		*p* < 0.0001	
DIF Mother	*r* = 0.67		*r* = 0.73	
	*p* < *0*.0001		*p* < *0*.0001	
DDF Mother	*r* = 0.46		*r* = 0.54	
	*p* = 0.002		*p* < 0.0001	
EOT Mother	*r* = 0.09		*r* = 0.21	
	*p* = 0.54		*p =* 0.19	
Total score Father		*r* = 0.37		*r* = 0.36
		*p* = *0*.02		*p* = 0.03
DIF Father		*r* = 0.32		*r* = 0.45
		*p =* 0.04		*p =* 0.005
DDF Father		*r* = 0.33		*r* = 0.21
		*p =* 0.04		*p* = 0.21
EOT Father		*r* = 0.18		*r* = 0.13
		*p =* 0.27		*p* = 0.43
**Toronto Structured Interview for Alexithymia (TSIA)**				
Total score Mother	*r* = 0.11		*r* = 0.15	
	*p* = 0.51		*p* = 0.35	
DIF Mother	*r* = 0.31		*r* = 0.25	
	*p* = 0.07		*p* = 0.13	
DDF Mother	*r* = 0.11		*r* = 0.11	
	*p* = 0.52		*p* = 0.49	
EOT Mother	*r* = 0.15		*r* = 0.21	
	*p* = 0.39		*p* = 0.22	
IMP Mother	*r* = 0.24		*r* = 0.17	
	*p* = 0.15		*p* = 0.32	
AA Mother	*r* = 0.23		*r* = 0.22	
	*p* = 0.16		*p* = 0.19	
OT Mother	*r* = 0.07		*r* = 0.35	
	*p* = 0.07		*p* = 0.04	
Total score Father		*r* = 0.13		*r* = 0.17
		*p* = 0.51		*p* = 0.36
DIF Father		*r* = 0.07		*r* = 0.25
		*p* = 0.7		*p* = 0.19
DDF Father		*r* = 0.38		*r* = 0.08
		*p* = 0.04		*p* = 0.68
EOT Father		*r* = 0.21		*r* = 0.03
		*p* = 0.28		*p* = 0.88
IMP Father		*r* = 0.05		*R* = −0.26
		*p* = 0.81		*p* = 0.18
AA Father		*r* = 0.28		*r* = 0.05
		*p* = 0.13		*p* = 0.81
OT Father		*r* = 0.11		*R* = −0.17
		*p* = 0.59		*p* = 0.41

DIF, difficulty identifying feelings; DDF, difficulty describing feelings (DDF); OT, externally-oriented thinking; IMP, imaginal processing; AA, affect awareness; OT: operative thinking. * α levels were corrected for multiple comparisons using hierarchical Sidak correction. We set an α_c_ < 0.006 for the correlation among STAI-Y and BDI-II with TAS-20 and TSIA total scores and an α_c_ < 0.009 for the correlation among STAI-Y and BDI-II with TAS-20 and TSIA subscales.

**Table 4:** Correlations between alexithymia scales, trait anxiety, and depression in parents of TD children.

TD Parent Group	STAI-Y	STAI-Y	BDI-II Mother	BDI-II Father
	Mother	Father		
**Toronto Alexithymia Scale (TAS-20)**				
Total score Mother	***r* = 0.45**		*r* = 0.36	
	***p* = 0.002**		*p* = 0.01	
DIF Mother	***r* = 0.37**		*r* = 0.28	
	***p* = 0.008**		*p* = 0.06	
DDF Mother	*r* = 0.35		*r* = 0.26	
	*p* = 0.01		*p* = 0.07	
EOT Mother	*r* = 0.11		*r* = 0.21	
	*p* = 0.43		*p* = 0.15	
Total score Father		*r* = 0.51		*r* = 0.31
		*p* < 0.0001		*p* = 0.03
DIF Father		*r* = 0.42		*r* = 0.4
		*p* = 0.003		*p* = 0.007
DDF Father		*r* = 0.45		*r* = 0.39
		*p* = 0.002		*p* = 0.007
EOT Father		*r* = 0.41		*r* = 0.11
		*p* = 0.004		*p* = 0.47
**Toronto Structured Interview for Alexithymia (TSIA)**				
Total score Mother	*r* = 0.43		*r* = 0.45	
	*p* = 0.01		*p* = 0.007	
DIF Mother	*r* = 0.29		*r* = 0.43	
	*p* = 0.1		*p* = 0.01	
DDF Mother	*r* = 0.28		*r* = 0.39	
	*p* = 0.11		*p* = 0.02	
EOT Mother	*r* = 0.42		*r* = 0.41	
	*p* = 0.01		*p* = 0.02	
IMP Mother	*r* = 0.19		*r* = 0.005	
	*p* = 0.28		*p* = 0.98	
AA Mother	*r* = 0.32		*r* = 0.45	
	*p* = 0.06		***p* = 0.008**	
OT Mother	*r* = 0.43		*r* = 0.35	
	*p* = 0.01		*p* = 0.04	
Total score Father		*r* = 0.42		*r* = 0.36
		*p* = 0.04		*p* = 0.09
DIF Father		*r* = 0.39		*r* = 0.43
		*p* = 0.06		*p* = 0.04
DDF Father		*r* = 0.4		*r* = 0.25
		*p* = 0.05		*p* = 0.25
EOT Father		*r* = 0.03		*r* = 0.02
		*p* = 0.87		*p* = 0.92
IMP Father		*r* = 0.21		*r* = 0.19
		*p* = 0.34		*p* = 0.37
AA Father		*r* = 0.51		*r* = 0.44
		*p* = 0.01		*p* = 0.03
OT Father		*r* = 0.15		*r* = 0.13
		*p* = 0.49		*p* = 0.54

DIF, difficulty identifying feelings; DDF, difficulty describing feelings (DDF); OT, externally-oriented thinking; IMP, imaginal processing; AA, affect awareness; OT, operative thinking. * α levels were corrected for multiple comparisons using hierarchical Sidak correction. We set an α_c_ <0.006 for the correlation among STAI-Y and BDI-II with TAS-20 and TSIA total scores and an α_c_ <0.009 for the correlation among STAI-Y and BDI-II with TAS-20 and TSIA subscales.
